# Intestinal perforation by an ingested foreign body*

**DOI:** 10.1590/0100-3984.2015.0127

**Published:** 2016

**Authors:** Gabriel Cleve Nicolodi, Cesar Rodrigo Trippia, Maria Fernanda F. S. Caboclo, Francisco Gomes de Castro, Wagner Peitl Miller, Raphael Rodrigues de Lima, Leandro Tazima, Jamylle Geraldo

**Affiliations:** 1 MD, Radiology Resident at Hospital São Vicente – Funef, Curitiba, PR, Brazil.; 2 MD, Radiologist and Preceptor at Hospital São Vicente – Funef, Curitiba, PR, Brazil.

**Keywords:** Intestinal perforation, Intestine, small, Foreign bodies, Abdomen, acute, Tomography, X-ray computed

## Abstract

**Objective:**

To identify the computed tomography findings suggestive of intestinal perforation by an ingested foreign body.

**Materials and Methods:**

This was a retrospective study of four cases of surgically proven intestinal perforation by a foreign body, comparing the computed tomography findings with those described in the literature.

**Results:**

None of the patients reported having ingested a foreign body, all were over 60 years of age, three of the four patients used a dental prosthesis, and all of the foreign bodies were elongated and sharp. In all four patients, there were findings indicative of acute abdomen. None of the foreign bodies were identified on conventional X-rays. The computed tomography findings suggestive of perforation were thickening of the intestinal walls (in all four cases), increased density of mesenteric fat (in all four cases), identification of the foreign body passing through the intestinal wall (in three cases), and gas in the peritoneal cavity (in one case).

**Conclusion:**

In cases of foreign body ingestion, intestinal perforation is more common when the foreign body is elongated and sharp. Although patients typically do not report having ingested such foreign bodies, the scenario should be suspected in elderly individuals who use dental prostheses. A computed tomography scan can detect foreign bodies, locate perforations, and guide treatment. The findings that suggest perforation are thickening of the intestinal walls, increased mesenteric fat density, and, less frequently, gas in the peritoneal cavity, often restricted to the point of perforation.

## Abstract (pt)

**Objetivo:**

Identificar os achados tomográficos sugestivos de perfuração intestinal por corpo estranho alimentar.

**Materiais e Métodos:**

Foram avaliados, retrospectivamente, quatro casos de perfuração intestinal por corpo estranho comprovados cirurgicamente, e comparados os achados tomográficos com os descritos na literatura.

**Resultados:**

Nenhum dos pacientes referiu a ingestão do corpo estranho, todos tinham mais de 60 anos, três usavam prótese dentária e todos os objetos eram alongados e pontiagudos. Os quatro apresentaram abdome agudo, o corpo estranho não foi identificado pela radiografia simples em nenhum deles, e os achados tomográficos sugestivos de perfuração foram espessamento das paredes da alça intestinal (nos quatro casos), densificação da gordura mesentérica (nos quatro casos), identificação do corpo estranho transfixando a parede intestinal (em três casos) e gás na cavidade peritoneal (em um caso).

**Conclusão:**

Perfuração intestinal é mais comum em casos de objetos pontiagudos e alongados, em que os pacientes, geralmente, não referem a ingestão, mas deve-se suspeitar em idosos que usam próteses dentárias. A tomografia permite detectar corpos estranhos, localizar a perfuração e orientar o tratamento. Os achados que sugerem perfuração são espessamento das paredes de um segmento intestinal, edema da gordura mesentérica e, menos frequentemente, gás na cavidade peritoneal, muitas vezes limitado ao ponto da perfuração.

## INTRODUCTION

Accidental ingestion of a foreign body together with food is a common clinical problem at emergency care facilities. Although most ingested foreign bodies pass through the gastrointestinal tract without consequences within one week^(1)^, in up to 1% of cases perforation occurs at some point in the gastrointestinal tract^(2)^. Perforation of the gastrointestinal tract is more common if the foreign body is elongated and sharp, like a fish bone, chicken bone, or toothpick, and occurs mainly in the small intestine, at points of physiological angulation or narrowing^(3)^. The clinical presentation is varied and often poses a diagnostic challenge. Patients generally do not report the ingestion of a foreign body, which delays the diagnosis and creates confusion with other diagnostic possibilities.

The objective of this study was to describe four cases of intestinal perforation by ingested foreign body and associate the tomography findings with those described in the literature.

## MATERIALS AND METHODS

We reviewed four cases of surgically confirmed intestinal perforation by ingested foreign body, all in the small intestine, treated in the emergency room between July 2012 and June 2013.

All patients presented with acute abdomen at the time of diagnosis. The perforation was by a fish bone in two cases, by a chicken bone in one case, and by a toothpick in one case. At presentation, none of the patients mentioned the possibility of foreign body ingestion. All cases were investigated by the routine protocol for acute abdomen and computed tomography (CT) of the abdomen.

## RESULTS

**Figure 1:**
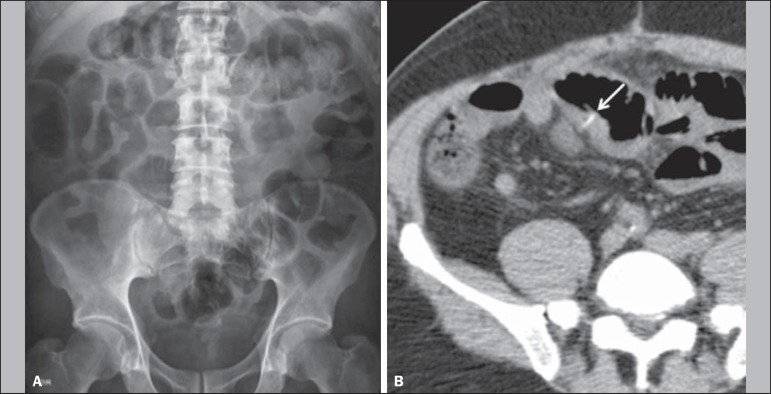
A 64-year-old female patient. **A:** The foreign body was not detected on routine X-rays. **B:** A CT scan of the abdomen showed a sharp foreign body (arrow), with the appearance of a fish bone, piercing the intestinal wall in the ileal segment within the pelvic cavity, accompanied by thickening of the intestinal wall and increased density of the adjacent mesenteric fat.

**Figure 2:**
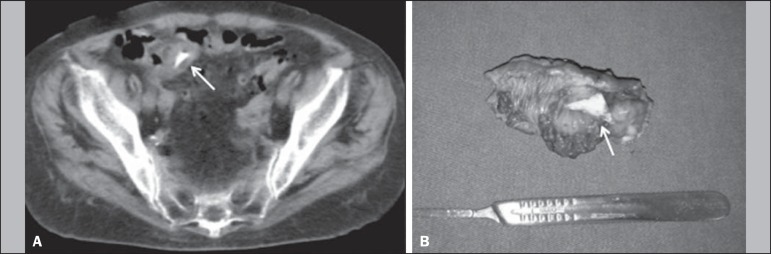
A 68-year-old male patient with severe abdominal pain. **A:** A CT scan of the abdomen showing a sharp foreign body (arrow) in the distal ileal segment, together with thickening of the intestinal wall. **B:** During the surgical procedure, a chicken bone fragment (arrow) was found to be piercing the intestinal wall and the affected intestinal segment was resected.

**Figure 3:**
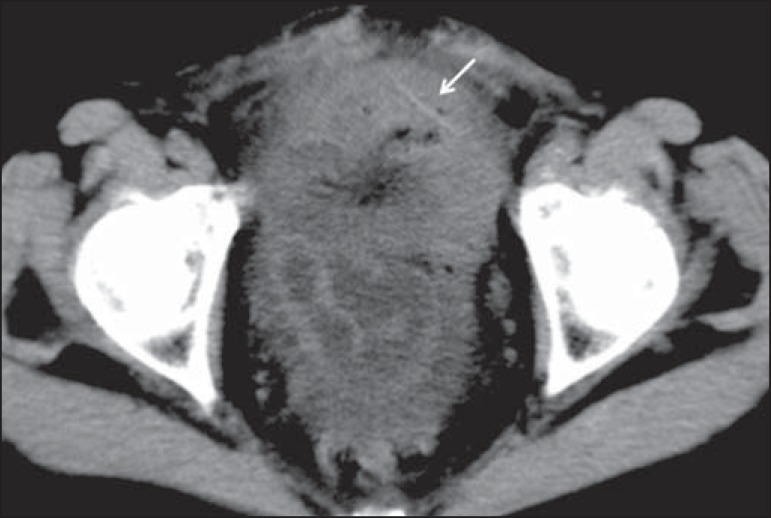
A 83-year-old female patient. A CT scan of the abdomen showing inflammatory impaction in the pelvic cavity. Surgery confirmed intestinal perforation by a toothpick, which was detected (retrospectively) as an image with a slightly higher density than the surrounding tissue, piercing the intestinal wall (arrow)

**Figure 4:**
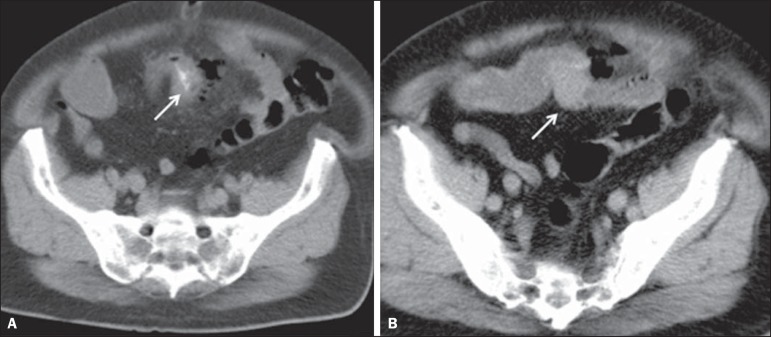
A 74-year-old male patient. A CT scan of the abdomen, showing a foreign body piercing the wall of the ileal loop in the pelvic region (**A**, arrow), with thickening of the intestinal wall, increased mesenteric fat density, and free gas in the peritoneal cavity, indicating intestinal perforation. Note also the nodular lesion with soft parts protruding into the intestinal lumen at the point of the foreign body impaction (**B**, arrow). Surgery revealed a neuroendocrine tumor in the intestinal wall, resulting in narrowing of the lumen, at the point of perforation by a fish bone.

Patient ages ranged from 64 to 83 years (mean, 71.5 years); two were male and two were female. In no case was the foreign body detected by routine X-ray (Figure 1). In three patients (two with perforation by a fish bone and one with perforation by a chicken bone), the foreign body, due to its calcium density, was identified on the CT scan, as were signs of intestinal perforation, including an image of the foreign body passing through the intestinal wall, distention of the intestinal lumen (with liquid stasis and thickening of the intestinal wall), increased mesenteric fat density, and free gas in the peritoneal cavity (Figure 2). In the case of intestinal perforation by a toothpick, the initial diagnosis was inflammatory impaction likely caused by intestinal perforation of unknown cause. Because of its low density, the toothpick was not identified in the initial CT scan. However, in a retrospective (postoperative) evaluation, it could be localized (Figure 3). Three of the patients used a dental prosthesis. In one of those patients, the foreign object perforated the intestine in an area of narrowing secondary to the presence of a neuroendocrine tumor in the intestinal wall (Figure 4).

All four patients presented thickening of the intestinal walls and increased mesenteric fat density. In three, the foreign body was identified passing through the intestinal wall, and gas in the peritoneal cavity was observed in only one.

## DISCUSSION

Intestinal perforation is typically caused by ingested foreign bodies that are sharp and elongated, such as fish bones, chicken bones, and toothpicks, being most common at points of physiological angulation or narrowing angulation or narrowing within the digestive tract^(3)^, up to 83% of all cases occurring in the ileal loops^(4)^. Fish bone, ingested accidentally, is the most common cause of perforation of the gastrointestinal tract^(5)^, although its incidence varies depending on the dietary habits of each population. In general, patients with intestinal perforation by an ingested foreign body present to emergency facilities with acute abdomen, which can include abdominal pain, nausea, vomiting, fever, peritonitis, abscess, fistula, intestinal obstruction, and gastrointestinal bleeding^(6)^. Patients typically do not report the ingestion of a foreign body, which, together with a clinical profile that is often confusing, can complicate and delay the diagnosis. Those that are most susceptible to foreign body ingestion include the elderly, denture wearers, alcoholics, and psychiatric patients^(6)^. Dentures reduces the tactile sensitivity of the palate, thus impairing the ability to sense small objects in the oral cavity^(2)^, and have been a factor reported in up to 80% of cases of accidental ingestion of a foreign body^(5)^. In the present study, three of the four patients were denture wearers.

Ingested foreign bodies are rarely detected on routine X-rays, because they usually have small dimensions and low radiopacity^(7)^, as well as because they are often obscured by intestinal gas. In one prospective study, involving 358 patients who had ingested a fish bone, routine X-ray of the abdomen showed a sensitivity of only 32%^(8)^.

In the evaluation of patients with acute abdomen, CT plays an important role, being considered a method with high sensitivity for the identification of intestinal perforation. The accuracy of CT in identifying the location of intestinal perforation is approximately 86%, making it an important tool for allowing surgeons to know the exact point of perforation, in order to plan the surgery^(9)^. It also has high sensitivity for detecting small, calcified foreign bodies such as fish bones and small fragments of chicken bone. In addition, CT can detect noncalcified foreign bodies (e.g., toothpicks) and identify areas of narrowing of the digestive tract, which predispose to impaction of the foreign body (e.g., inflammatory or neoplastic areas of stenosis). With respect to toothpicks, previous studies have shown that their attenuation can vary in function of the amount of air and fluid within the wood. When ingested, a toothpick tends to be dry and predominantly filled with air, having a lower attenuation coefficient, which, due to the absorption of fluids, increases after a few days^(10,11)^.

One of the patients evaluated here presented intestinal perforation by a fish bone in an area of narrowing caused by a neuroendocrine tumor in the intestinal wall. Various studies have established CT as a method of choice for the investigation of pneumoperitoneum^(12)^, which is an important factor in determining the sensitivity of the method for identifying perforation of the hollow viscera. With the advent of multislice CT and the ability to make finer slices, the sensitivity of the method improved^(5)^. The use of oral contrast during CT can make it more difficult to detect a radiopaque foreign body. The region of intestinal perforation can be identified on CT scans as an intestinal segment with thickened walls, increased mesenteric fat density, and gas in the peritoneal cavity, the last often limited to the point of perforation. Because intestinal perforation is caused by impaction and progressive erosion of the foreign body in contact with the intestinal wall, the perforation site is typically covered with fibrin, omentum, and other intestinal loops, thus limiting the passage of large amounts of gas into the peritoneal cavity^(13)^.

In our series of cases, the most common indicator of intestinal perforation was the finding of an intestinal segment with a thickened wall at the point of foreign body impaction, together with increased mesenteric fat density. In one case, we found gas in the peritoneal cavity adjacent to the point of perforation, which is consistent with data in the literature. Intestinal obstruction, which is a rare finding, was observed in one of the cases evaluated here. That can be explained by the fact that perforation was not suspected, resulting in a longer period of evolution, which promoted obstruction and inflammatory impaction around the perforation site. The CT findings are indistinguishable from those of intestinal obstruction by other causes, such as blockages or tumors, resulting in distention and liquid stasis in the upstream intestinal loops (diameter > 2.5 cm), with identification of an area of transition between the dilated loops proximal to and the collapsed loops distal to the point of obstruction^(14)^. In addition to the obstruction, gastrointestinal bleeding can, in rare cases, occur secondary to foreign bodyinduced erosion of the intestinal wall into a feeding blood vessel. Acute appendicitis and Meckel's diverticulum caused by impaction of a foreign body are quite rare, although some cases have been described in the literature^(15,16)^.

The treatment strategy depends on the location of foreign body in the digestive tract and the presence or absence of complications such as perforation, hemorrhage, and obstruction. Foreign bodies located in the esophagus or stomach are preferentially removed endoscopically, whereas those located in the small intestine are surgically treated with segmental resection of the affected loop^(17,18)^.

## CONCLUSION

Although the accidental ingestion of a foreign body is a common event, intestinal perforation is an unusual finding. However, when it occurs, it manifests as acute abdomen and constitutes a diagnostic challenge in emergency medicine. CT has contributed significantly to its diagnosis and is the best imaging method for identifying foreign bodies with minimal radiopacity, allowing the exact location of the perforation site to be determined and the surgical treatment to be planned reliably. The imaging findings that suggest intestinal perforation are an intestinal segment with thickened walls, increased mesenteric fat density, and, less often, gas in the peritoneal cavity, usually restricted to the perforation site. In most cases, the patient does not report the possibility of ingestion of foreign matter, however, the diagnosis should be suspected in cases of acute abdomen of unknown cause in elderly patients and in denture wearers.
